# A Novel Mouse Model Unveils Protein Deficiency in Truncated CDKL5 Mutations

**DOI:** 10.1007/s12264-024-01346-4

**Published:** 2025-03-05

**Authors:** Xue Feng, Zi-Ai Zhu, Hong-Tao Wang, Hui-Wen Zhou, Ji-Wei Liu, Ya Shen, Yu-Xian Zhang, Zhi-Qi Xiong

**Affiliations:** 1 Institute of Neuroscience, CAS Center for Excellence in Brain Science and Intelligence Technology, Chinese Academy of Sciences, Shanghai, 200031 China; 2 School of Life Science and Technology, ShanghaiTech University, Shanghai, 201210 China; 3 University of Chinese Academy of Sciences, Beijing, 100049 China; 4 Shanghai Mental Health Center, Shanghai Jiao Tong University School of Medicine, Shanghai, 200030 China; 5 Shanghai Center for Brain Science and Brain-Inspired Intelligence Technology, Shanghai, 201210 China; 6 School of Future Technology, University of Chinese Academy of Sciences, Beijing, 100049 China

**Keywords:** *Cdkl5*, Truncating mutations, CDKL5 deficiency disorder, Nonsense-mediated RNA decay

## Abstract

Mutations in the cyclin-dependent kinase-like 5 gene (*CDKL5*) cause a severe neurodevelopmental disorder, yet the impact of truncating mutations remains unclear. Here, we introduce the *Cdkl5*^*492stop*^ mouse model, mimicking C-terminal truncating mutations in patients. 492stop/Y mice exhibit altered dendritic spine morphology and spontaneous seizure-like behaviors, alongside other behavioral deficits. After creating cell lines with various *Cdkl5* truncating mutations, we found that these mutations are regulated by the nonsense-mediated RNA decay pathway. Most truncating mutations result in CDKL5 protein loss, leading to multiple disease phenotypes, and offering new insights into the pathogenesis of CDKL5 disorder.

**Supplementary Information:**

The online version contains supplementary material available at 10.1007/s12264-024-01346-4.

## Introduction

Mutations in the X-linked cyclin-dependent kinase-like 5 gene (*CDKL5*) are associated with CDKL5 deficiency disorder (CDD [MIM300203]) [1], a neurodevelopmental disease characterized by early infantile epileptic encephalopathy, autism spectrum disorders, and intellectual disability [2–6]. CDKL5 is a serine/threonine kinase that shares homology with cyclin-dependent kinases and mitogen-activated protein kinases [2]. It plays a crucial role in proper brain development [7–10], with its highest expression during early postnatal development and remaining at elevated levels throughout adulthood [11]. However, the exact mechanisms underlying the pathogenesis of CDD remain unclear, and effective therapies are lacking.

The reported missense mutations in CDKL5 mostly impair the N-terminal catalytic domain, leading to a loss of kinase activity [12]. Separately, frameshift and nonsense variants scattered throughout the gene frequently generate premature termination codons, which are commonly found in CDD patients [5, 13]. Despite these findings, the *in vivo* effects of C-terminal truncating mutations remain poorly understood. Whether these mutations lead to loss-of-function or gain-of-function effects, as well as the existence of truncated CDKL5 proteins, requires further investigation.

To investigate these questions, we generated a *Cdkl5*^*492stop*^ mouse model, mimicking C-terminal truncating mutations in patients. These mice exhibited altered spine morphology across brain regions and behavioral deficits such as reduced sociability, impaired motor coordination, and increased anxiety, consistent with other *Cdkl5* knockout models [7, 14, 15]. Notably, noninvasive video recording and electroencephalographic (EEG) analysis confirmed spontaneous seizure-like behaviors in hemizygous *Cdkl5* knockout mice.

Furthermore, our study revealed that truncated CDKL5 mutants undergo protein loss due to regulation by the nonsense-mediated mRNA decay (NMD) pathway. Premature termination codons (PTCs) upstream of amino-acid 763, found in over a third of CDD patients [5, 16], lead to CDKL5 protein degradation and associated functional deficits. Insights from *Cdkl5*^*492stop*^ mice and cell lines highlight the mechanism of CDKL5 deletion through protein loss, providing valuable tools for translational and pathogenic studies of CDD.

## Materials and Methods

### Mouse Model Generation

The *Cdkl5*^*492stop*^ mouse model was generated using CRISPR/Cas9 technology. Single guide RNAs targeting the *Cdkl5* gene were designed (guide RNA target site: 5′-ACACCTTCTCAGTCCAAAAGA-3’) and injected into fertilized eggs from C57BL/6J mice. The injected zygotes were then implanted into pseudopregnant female mice. Pups were screened for the presence of the *Cdkl5* truncating mutation by PCR and Sanger sequencing. Positive founder mice were bred to establish a *Cdkl5*^*492stop*^ colony. Genotyping was applied using PCR-based sequencing with the following primers:

Forward: 5′- TTTGGCCTTTGTCCTGTAGGATGG -3′

Reverse: 5′- ATACTCCTTCCTTCCCTGAGCC -3′

*Cdkl5*^*492stop/+*^ females were crossed with C57BL/6J wild-type (WT) males to generate *Cdkl5*^*492stop/Y*^ and *Cdkl5*^*wt/Y*^ mice. The *Cdkl5* knockout (KO) mice [14] were *Cdkl5*^*flox/flox*^ mice bred with Nestin-driven Cre-mediated mouse lines (JAX 003771).

The mice were housed in groups of six littermates per cage, maintained at a temperature of 22 ± 1°C, under a 12 h light-dark cycle, with *ad libitum* access to food and water. All experimental procedures were approved by the Institutional Animal Care and Use Committee (No. NA-009-2022) of the Institute of Neuroscience, Chinese Academy of Sciences, and conducted in accordance with the Society for Neuroscience guidelines.

### Behavioral Assays

All behavior studies were conducted in a blinded manner. Mice were given a habituation period of 30 min in the testing room prior to each session. The behavioral tests were performed on adult male 492stop/Y mice and WT littermates aged 10-14 weeks.

#### Rotarod

The rotarod test was used to assess motor balance and motor learning. Each mouse was placed on a rotating rod (3 cm diameter) that accelerated from 5 rpm to 40 rpm over 300 s (Ugo Basile, Comerio, Italy). Three trials were conducted with at least a 15-min interval between each trial. Mice underwent priming for adaptation on the first day and were recorded over the following 5 days. The maximum test duration was 400 s on day 5 and 500 s on the last day. Fall latency during the three trials was recorded, and the best score was used for analysis.

#### Wire Hang

The wire hang test evaluated muscle strength and motor coordination. A horizontal wire (0.2 cm diameter, 54.5 cm length) was suspended between two rods and lifted off a padded surface. Mice were initially hung from the wire using both forelimbs.

#### Gait Analysis

The CatWalk XT system was used for gait analysis to compare the walking patterns of transgenic mice with WT control mice. Mice walked across a track (50 cm long and 10 cm wide) within an enclosed box. The hind and front paw prints were recorded and analyzed using the CatWalk XT software.

#### Open Field

The open field test was conducted to assess the anxiety levels. Each mouse was placed in the center of a 40 cm × 40 cm × 30 cm open field device and allowed to freely explore for 30 min; data were acquired and analyzed using EthoVision XT software.

#### Light-Dark Transition Test

The light-dark test involved presenting mice with a conflict situation between the desire to explore and the stress response to the unknown. The test apparatus consisted of a compartment divided into one-third dark and two-thirds light compartments, with overall dimensions of 46 cm × 27 cm × 30 cm. Mice were placed in the dark side and allowed to move between the two chambers for 15 min. The time spent in each chamber was recorded using the EthoVision XT software.

#### Three-Chambered Social Approach Task

The social approach assay was applied using a three-chambered apparatus as previously described [17]. Mice underwent two days of habituation to the chambers and became familiar with a stimulus mouse placed in the chamber at one end (social chamber), while the chamber at the other end remained empty (non-social chamber). During the test, the mouse being tested was placed in the middle chamber, and the time spent exploring each end chamber was recorded for 10 min; data were acquired and analyzed using the EthoVision XT software. Mice showing a clear chamber preference were excluded from the analysis.

#### Fear Conditioning

Fear conditioning tests were conducted to assess learning and memory. Before the test day, each mouse had a 5-min habituation period in the fear box. On the test day, they were exposed to a 90-dB white noise (CS) for 30 s, followed by a mild footshock (2 s, 0.5 mA, US). Two more CS-US pairings were given at 1-min intervals. The context test was applied 24 h later, and altered context cue tests were done 48 h after conditioning with the same sound. Video captured at one frame per second measured the mouse's movement, and freezing behavior was determined based on a predefined threshold. Data were analyzed using FreezeFrame 4.0 software.

#### Nesting

We assessed nesting impairment, a phenotype related to home-cage social behavior reported in Autism Spectrum Disorder and Rett Syndrome mouse models. About 1 h before the dark period, mice were individually placed in nesting cages containing a nestlet, a 5-cm square of pressed cotton wadding. The proportion of the torn nestlet and the quality of the nest were assessed the following morning [18].

### Video, EMG, and EEG Recordings

For the *in vivo* tests, EEG and electromyographic (EMG) electrodes were implanted [19]. After a recovery period of at least three days, the mice were habituated to the recording setup for two consecutive days. EEG and EMG were simultaneously recorded from freely-moving and head-restrained mice. On the following day, the mice were videotaped for one day. EEG recordings were amplified ×100 (Model 1800, A-M Systems, WA, USA), with a high-pass filter at 0.1 Hz and a low-pass filter at 100 Hz. The signals were then digitized at 1 kHz using Spike2 software (Micro1401 mkII, Cambridge Electronic Design).

### Analysis of Dendritic Spine Morphology

Golgi-Cox staining was used to analyze dendritic spine morphology in the brains of 492stop/Y mice and WT controls, using the FD Rapid GolgiStainTM Kit (FD NeuroTechnologies, Inc., Columbia, USA) according to the manufacturer’s instructions. Sections were imaged under a confocal microscope, and dendritic spine density and morphology were analyzed using ImageJ software.

### Cell Culture and Transfection

Hippocampal pyramidal neurons were extracted from P0 C57BL/6J mice as previously described [20] and cultured in a neurobasal medium supplemented with 1% B27 (Gibco, NY, USA) and 10 mmol/L GlutaMax (Gibco). HEK293T cells were grown in Dulbecco’s modified Eagle’s medium (Gibco) supplemented with 10% fetal bovine serum (Gibco). Mouse embryonic stem cells were grown in Dulbecco’s modified Eagle’s medium (Merck Millipore, MA, USA) supplemented with 15% fetal bovine serum (Gibco), 1000 U/mL mouse leukemia inhibitory factor (Merck Millipore), 2 mmol/L GlutaMax (Gibco), 1% penicillin/streptomycin (Gibco), 0.1 mmol/L 2-mercaptoethanol (Merck Millipore), 0.1 mmol/L non-essential amino acids (Gibco), 1 μmol/L PD0325901 (Selleck Chemicals, TX, USA), and 3 μmol/L CHIR99021 (Selleck Chemicals). Neurons were transfected using Lipofectamine 2000 (Invitrogen, CA, USA), and cell lines were transfected using Lipofectamine 3000 (Invitrogen) following the manufacturer’s instructions. The cells were treated with 50 μmol/L NMDI14 (MedChemExpress, NJ, USA).

### Image Analysis

Confocal images of dendritic spines were acquired using a confocal microscope (Nikon A1, Tokyo, Japan) with a 63×/1.4 NA oil immersion objective and 3× optical zoom. Images were captured at 1 µm intervals and analyzed in a blinded manner using ImageJ software.

### DNA Constructs

PCR products corresponding to the murine CDKL5105 isoform (GenBank accession number NM_001024624) were cloned into the pCAG-tag2B vector. Plasmid-based RNAi constructs were generated using the pSuper vector (Oligoengine, WA, USA). The sequences of the UPF1 shRNAs used were as follows:

shUPF1-9: GTATATTTACCACAAGCTGCT

shUPF1-10: GATGCAGTTCCGTTCCATC

All constructs were validated through sequencing.

### Lysate Preparation

Fresh brain tissue or cultured cells were homogenized in lysis buffer consisting of 20 mmol/L Tris-HCl (pH 7.4), 300 mmol/L NaCl, 1% Triton X-100, 1 mmol/L EDTA, 1 mmol/L EGTA, and 10% glycerol, along with protease inhibitors (1 mmol/L phenylmethylsulfonyl fluoride and protease cocktail) and phosphatase inhibitors (1 mmol/L NaF and 1 mmol/L Na3VO4). Homogenization was applied using a frozen ball mill (Shanghai Jingxin Industrial Development Co., Shanghai, China). The lysates were incubated at 4°C for 30 min and then centrifuged at 14,000 × g for 15 min; the protein concentration of the supernatants was determined using a BCA Protein Assay (Epizyme Biotech, Shanghai, China).

### Western Blotting

Protein samples were separated by SDS-PAGE and transferred onto a PVDF membrane (Merck Millipore). Membranes were probed with primary antibodies and visualized using enhanced chemiluminescence, then the intensities were measured using ImageJ software (Fiji). The following antibodies were obtained from commercial sources: CDKL5 (SA145, MRC PPU, Scotland, UK); CDKL5 (ABS402, Merck Millipore); EB2 (ab45767, Abcam, Cambridge, UK); EB2 pS222 (00117739, Covalab, Grenoble, France); UPF1 (#12040, Cell Signaling Technology, MA, USA).

### Real-Time PCR

Total mRNA was extracted from mouse brain tissue and cultured neurons. TRIzol (Invitrogen) and the EZ-press RNA Purification Kit (EZBioscience, OH, USA) were used for mRNA extraction. First-strand cDNA synthesis was performed using the iScript cDNA Synthesis Kit (BioRad, CA, USA). Real-time PCR was conducted using the LightCycler RNA Master SYBR Green I kit (Roche, Basel, Switzerland), and gene expression was analyzed with GAPDH or RPL19 as the reference gene in three biological replicates. The fold change was calculated as the ΔΔCT between the reference gene and target gene transcript levels, normalized to the mock-treated relative target gene transcript level. The primer sequences used were as follows:

CDKL5-F: CGTCCTCACAGGCATTCCAT

CDKL5-R: TCTCCAGGCTGGGGTGATAA

GAPDH-F: ATCCCAGAGCTGAACGGGAAGC

GAPDH-R: TTGGGGGTAGGAACACGGAAGG

RPL19-F: CTGAAGGTCAAAGGGAATGTG

RPL19-R: GGACAGAGTCTTGATGATCTC

UPF1-F: AGAACATCTTCGGGCCACTG

UPF1-R: AGGTCTTCAGTGCACTCTGC

### Experimental Design and Statistical Analysis

All data are presented as the mean ± SEM. Experimenters were blinded to the genotypes of the animals during image capture and analysis. Statistical analysis was applied using GraphPad Prism 5. Two groups were compared using a two-tailed unpaired Student’s *t*-test. Multiple comparisons between two groups were analyzed using a two-way ANOVA followed by Sidak’s *post-hoc* test. Multiple groups were compared using a one-way ANOVA followed by Tukey’s *post-hoc* test. Statistical significance was defined as *P < 0.05, **P < 0.01, ***P < 0.001, and ****P < 0.0001.

## Results

### Generation of the *Cdkl5*-Truncated Mouse Model

**Fig. 1:**
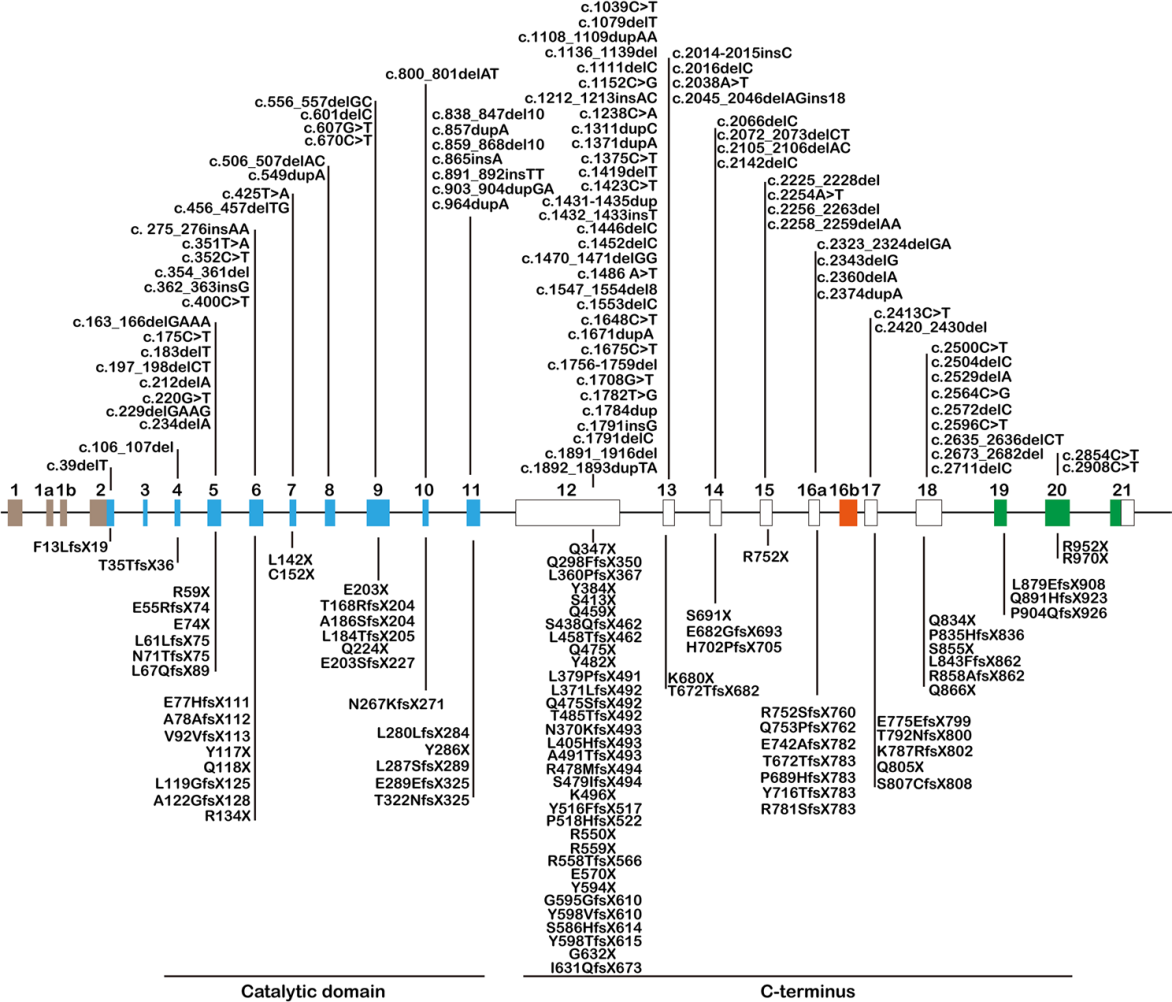
Pathogenic CDKL5 truncating mutations. Truncating mutations in CDKL5 are depicted along the gene, showing both the cDNA and amino-acid nomenclature in the CDKL5 gene (hCDKL5115 isoform as background). These mutations include deletions, insertions, frameshifts, and nonsense mutations. Examples include: c.39delT, F13LfsX19; c.1079delT, L360PfsX367; c.1238C>A, S413X; c.2014-2015insC, T672TfsX682 [21]; c.106_107del, T35TfsX36 [6]; c.163_166delGAAA, E55RfsX74 [23, 24]; c.175C>T, R59X [31, 32]; c.183delT, L61LfsX75; c.2854C>T, R952X [3]; c.197_198delCT, L67QfsX89 [36]; c.212delA, N71TfsX75; c.1108_1109dupAA, N370KfsX493; c.1553delC, P518HfsX522; c.1891_1916del, I631QfsX673 [40]; c.220G>T, E74X; c.351T>A, Y117X; c.456_457delTG, C152X; c.506_507delAC, T168RfsX204; c.556_557delGC, A186SfsX204; c.670C>T, Q224X; c.1547_1554del8, Y516FfsX517; c.1671dupA, R558TfsX566; c.2258_2259delAA, Q753PfsX762; c.2413C>T, Q805X; c.2420_2430del, S807CfsX808 [38]; c.229delGAAG, E77HfsX111; c.352C>T, Q118X; c.425T>A, L142X; c.865insA, E289EfsX325; c.2016delC, T672Tfs783 [4]; c.234delA, A78AfsX112; c.891_892 insTT, Q298FfsX350; c.1111delC, L371LfsX492; c.1791insG, Y598VfsX610; c.2360delA, K787RfsX802 [35]; c.275_276insAA, V92VfsX113; c.1784dup, G595GfsX610; c.2105_2106delAC, H702PfsX705 [26]; c.354_361del, L119GfsX125; c.549dupA, L184TfsX205; c.601delC, E203SfsX227; c.1212_1213insAC, L405HfsX493; c.1419delT, Q475SfsX492; c.1431-1435dup, S479IfsX494; c.1486 A>T, K496X; c.1756-1759del, S586HfsX614; c.2142delC, Y716TfsX783; c.2256_2263del, R752SfsX760; c.2529delA, L843FfsX862; c.2673_2682del, Q891HfsX923 [43]; c.362-363insG, A122GfsX128; c.2254A>T, R752X [33]; c.400C>T, R134X [6, 37, 39]; c.607G>T, E203X; c.1708G>T, E570X [22]; c.800_801delAT, N267KfsX271; c.1311dupC, S438QfsX462; c.1892_1893dupTA, G632X; c.2045_2046delAGins18, E682GfsX693; c.2323_2324delGA, E775EfsX799 [4, 27]; c.838_847del10, L280LfsX284; c.2343delG, R781SfsX783 [28]; c.857dupA, Y286X; c.859_868del10, L287SfsX289; c.1371dupA, L458TfsX462; c.1446delC, Y482X; c.1452delC, T485TfsX492 c.1470_1471delGG, A491TfsX493; c.1782T>G, Y594X; c.1791delC, Y598TfsX615; c.2038A>T, K680X; c.2072_2073delCT, S691X; c.2374dupA, T792NfsX800; c.2564C>G, S855X; c.2572delC, R858AfsX862; c.2711delC, P904QfsX926 [6]; c.903_904dupGA, L302DfsX350 [29]; c.964dupA, T322NfsX325; c.2066delC, P689HfsX783 [23]; c.1039C>T, Q347X [21, 38]; c.1152C>G, Y384X [39, 40]; - c.1136_1139del, L379PfsX491 [44]; c.1375C>T, Q459X [6, 35]; c.1432_1433insT, R478MfsX494 [25]; c.1648C>T, R550X [6, 29, 38]; c.1423C>T, Q475X [45]; c.1675C>T, R559X [6, 21]; c.2225_2228del, E742AfsX782 [34]; c.2500C>T, Q834X [4, 41]; c.2504delC, P835HfsX836 [6, 12]; c.2596C>T, Q866X [6, 42]; c.2635_2636delCT, L879EfsX908 [4, 6, 24, 27]; c.2908C>T, R970X [30].

We compiled data from >100 CDD patients, drawing from previous reports [3, 4, 6, 12, 21–42] and databases [43–45]. The schematic (Fig. 1) illustrates various truncating mutations, including nonsense mutations, small and large deletions/insertions, and frameshifts. These mutations were randomly distributed across the *Cdkl5* gene.

**Fig. 2:**
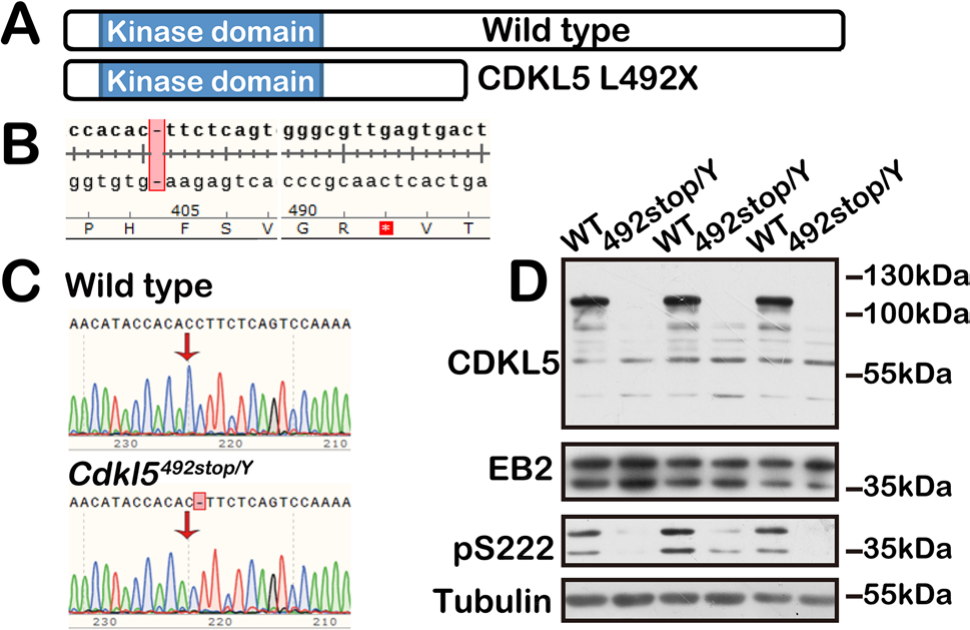
Generation of the 492stop/Y mouse model. **A** Schematic of the CDKL5 protein in the WT controls and with the L492X mutation. **B** Targeting strategy: Introduction of a C deletion at position 1471 nt results in a frameshift and a premature termination codon at amino-acid 492. **C** Sequencing of PCR products from genomic DNA in 492stop/Y mice and WT controls. **D** Western blots showing CDKL5 loss in 492stop/Y mice (*n =* 3). The full-length and predicted length (54 kDa) of CDKL5 protein are absent. Ser222, the phosphorylation site of EB2.

To investigate the pathogenic mechanism of C-terminal truncating mutations in CDD, we generated a novel mouse model. We selected the amino-acid 492 from exon 12, a high-frequency mutation site within the prevalent exon in CDD patients (Figs 1, 2A). A C deletion at position 1471 nt (NM_001024624) induced a frameshift, leading to premature termination at amino-acid 492 (Fig. 2A, B), establishing the *Cdkl5*^*492stop*^ mouse line, referred to as *Cdkl5* 492stop or 492stop (Fig. 2C).

Western blot analysis of forebrain extracts from 492stop/Y mice demonstrated the complete loss of CDKL5 immunoreactivity, both in the predicted length and full length of the protein (Fig. 2D). Immunohistochemical staining of EB2, a known CDKL5 substrate [46], showed significantly reduced phosphorylation at Ser222, confirming the loss of CDKL5 function. Therefore, CDKL5 protein expression and function were completely abolished in 492stop/Y mice, similar to the knockout mice described in previous studies.

### Motor Impairments and Increased Anxiety in 492stop/Y Mice

**Fig. 3:**
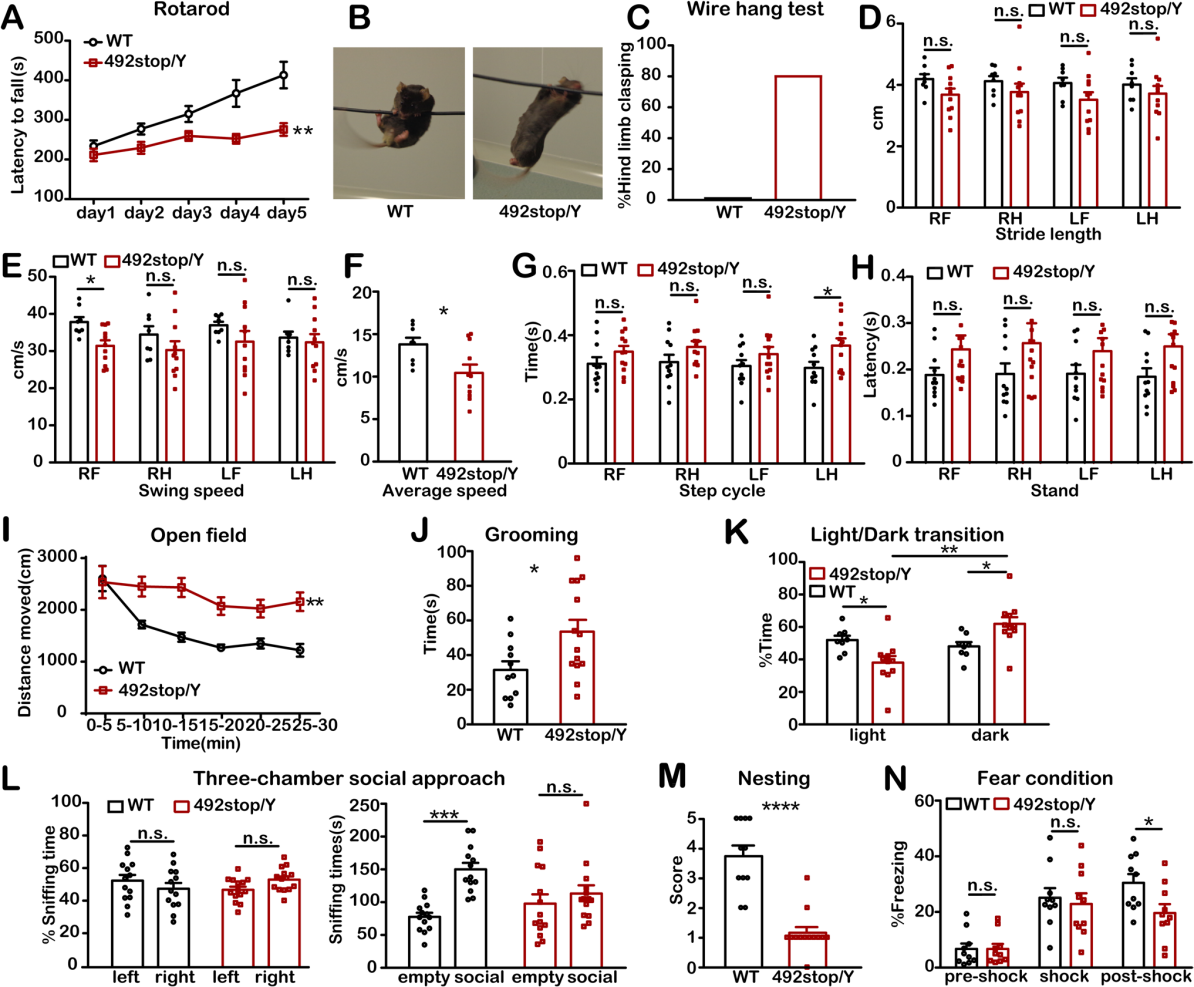
Behavioral phenotyping of 492stop/Y mice. **A** The Rotarod assay measures the latency of mice to fall from an accelerating rotating rod. Fall latency is lower in 492stop/Y mice (*n =* 10) than in WT littermates (*n =* 11), indicating impaired motor coordination. **P <0.01, two-way ANOVA, Sidak's multiple comparisons test. **B, C** The wire hang test. **B** Image of a 492stop/Y mouse hanging from the wire shows abnormal limb clasping. **C** 0/8 of WT mice and 8/10 (80%) of 492stop/Y mice exhibit limb clasping. **D**-**H** Gait analysis. RF: right forelimb; RH: right hindlimb; LF: left forelimb; LH: left hindlimb. The statistics record the stride length (**D**), swing speed (**E**), average speed (**F**), step cycle (**G**), and stand (**H**). *P <0.05, n.s.: no significant difference, unpaired two-tailed Student’s *t*-test. **D**-**F** WT littermates: *n =* 8, 492stop/Y mice: *n =* 11; **G**, **H** WT littermates: *n =* 11, 492stop/Y mice: *n =* 12. **I** Open field test, recording the locomotion of mice during a 30-min exploration in a novel open environment. WT littermates: *n =* 11, 492stop/Y mice: *n =* 14, **P <0.01, two-way ANOVA, Sidak's multiple comparisons test. **J** Grooming assay. Recordings of each mouse’s grooming time within 30 min; WT littermates: *n =* 11, 492stop/Y mice: *n =* 14, *P <0.05, unpaired two-tailed Student’s *t*-test. **K** Light-dark transition test. The exploration time of the mice in the dark box and light box up to the total time. Light: light box; dark: dark box. WT littermates: *n =* 8, 492stop/Y mice: *n =* 11. *P <0.05, **P <0.01, two-way ANOVA, Sidak's multiple comparisons test. **L** Three-chambered social approach assay. Data from mice with left/right blank box preferences were excluded. Left: left empty chamber; right: right empty chamber. WT: *n =* 13, 492stop/Y: *n =* 14, n.s. no significant difference. The exploration times of mice in the social box and empty box. Empty: empty chamber; social: social chamber; ***P <0.001, n.s.: no significant difference, two-way ANOVA, Sidak's multiple comparisons test. **M** Nesting assay. Summary of the level of mouse nesting behavior. WT littermates: *n =* 11, 492stop/Y mice: *n =* 13, ****P <0.0001, unpaired two-tailed Student’s *t*-test. **N** Fear conditioning paradigm, measuring the freezing time of WT (*n =* 10) and 492stop/Y (*n =* 10) mice before and after footshock. *P <0.05, n.s.: no significant difference, two-way ANOVA, Sidak's multiple comparisons test. All data are presented as the mean ± SEM.

Consistent with previous reports on *Cdkl5*-KO [7–9, 14], adult 492stop/Y mice displayed various behavioral phenotypes, including motor impairments and increased anxiety. We assessed motor coordination and learning using the rotarod test. While both WT and 492stop/Y mice improved in staying on the accelerating rod over the 5-day trial, the 492stop/Y mice exhibited only a slight increase in fall latency and impaired performance compared to WT littermates (Fig. 3A). Similar motor deficits were found in the wire hang test, where most 492stop/Y mice displayed hindlimb clasping within 20 s, preventing them from climbing the wire like the WT mice (Fig. 3B, C). Gait analysis also revealed coordination disturbances in 492stop/Y mice (Fig. 3D-H).

We next assessed anxiety-related behaviors in 492stop/Y mice using several behavioral tests, including the open field, light-dark transition, and grooming tests. In the open field test, 492stop/Y mice exhibited increased locomotion, as well as a longer duration and higher frequency of grooming than WT littermates (Fig. 3I-J). This suggests that the mutant mice had difficulty adapting to a new environment and showed hyperactivity even after a 30-min habituation period, indicating increased anxiety. Similarly, in the light-dark transition test, 492stop/Y mice displayed a significant preference for the dark chamber (62%), avoiding the unknown chamber and exhibiting less exploration than WT littermates (Fig. 3K). These findings are consistent with the impaired coordination and increased anxiety reported in other *Cdkl5*-KO mouse models [47].

### Autistic-Like Social Behavior and Learning Impairments in 492stop/Y Mice

To investigate autistic-like behavior, we used the three-chambered social approach task to assess social interaction in 492stop/Y mice. Unlike their WT littermates, which showed an interest in social interaction and spent more time in the social chamber (67%), the 492stop/Y mice spent equal time in the social and empty chambers (Fig. 3L). Mice showing a strong preference for the empty chamber on one side were excluded from the analysis. In addition, we found severe nesting deficits in 492stop/Y mice, further indicating impaired social behaviors (Fig. 3M).

Intellectual disability is a key feature of CDD [3, 48]. We assessed learning and memory in 492stop/Y mice using the fear conditioning test. Both genotypes exhibited similar freezing levels before and after footshock, but during re-exposure without footshock, 492stop/Y mice had a 36% reduction in freezing time compared to WT mice, indicating impaired fear-related learning and memory (Fig. 3N). These findings indicate significant deficits in social behavior, learning, and memory in 492stop/Y mice, alongside motor impairments and increased anxiety.

### Spontaneous Seizures and High Mortality in *Cdkl5* 492stop/Y Mice

Spontaneous seizures, a prominent phenotype in CDD patients, are reported to be increased in heterozygous female CDKL5-deficient mice [49, 50] and mice with glutamatergic forebrain-specific *Cdkl5* deletion [19]. To study seizure activity, we made long-term continuous video recordings over several months of 15 492stop/Y mice. From this, generalized seizure-like behaviors were not detected in the daily home cage activities, except for those leading to death. Of 11 recorded deaths, 5 were clearly captured on video. Two nearly 8-month-old mice died from spontaneous seizures in their home cages. Prior to death, these mice experienced a tonic-clonic seizure characterized by Straub tail, head nodding, rapid circling, jumping, and subsequent muscle relaxation of the tail and limbs (Video in Supplementary). No other abnormalities were noted in the 48 h of video preceding the fatal event. The remaining three mice died from unknown causes, possibly related to asphyxiation due to epilepsy [51].

**Fig. 4:**
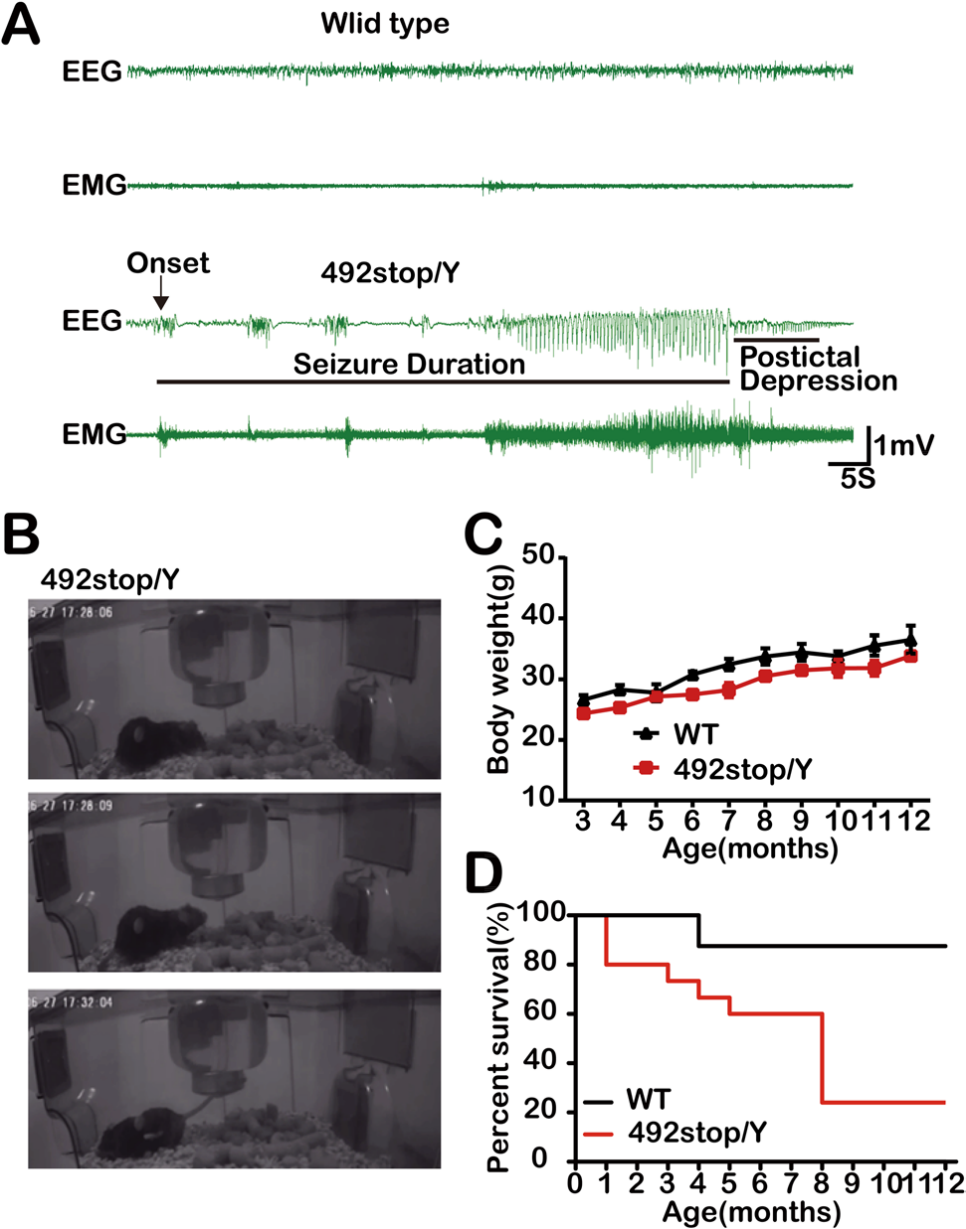
Spontaneous seizures in 492stop/Y mice. **A**, **B** Spontaneous seizures in Z22, a 13-month-old 492stop/Y mouse. **A** Representative electroencephalographic (EEG) and electromyographic (EMG) traces of epileptiform discharges in a 492stop/Y mouse. The arrow indicates the beginning of the seizure. **B** Image of a 492stop/Y mouse in the home-cage environment exhibiting seizure-like activity, including head nodding, forelimb clonus, and myoclonic jerks. **C** Statistical analysis of body weight in WT (*n =* 13) and 492stop/Y mice (*n =* 23) of different ages. Error bars represent the mean ± SEM. **D** Survival curves of 492stop/Y mice (*n =* 15) and WT controls (*n =* 8) at different ages. None of the WT mice show spontaneous seizures.

Previous studies have commonly reported spontaneous epileptic seizures in aged mice [19, 49, 50]. To investigate seizure events in 492stop/Y mice, we conducted 24-h EEG and EMG recordings on mice >1 year old. Fortunately, we detected typical epileptic wave patterns in two mice. In one 13-month-old 492stop/Y mouse (Fig. 4A), high-amplitude EEG spikes were recorded alongside EMG fluctuations during the awake state. Each epileptic episode lasted ~5 min, with a marked increase in wave amplitude occurring regularly at intervals of several hours throughout the day. Concurrent video recording revealed typical and progressive seizure-like activity in the mutant mouse, including Straub tail and myoclonic jerks (Fig. 4B). The size, duration, and frequency of the seizure events were consistent with the EEG results. Therefore, unlike other *Cdkl5*-KO mouse models, 492stop/Y mice exhibited spontaneous seizure activity characterized by lethality, abruptness, and low frequency.

In addition, the 492stop/Y mice were prone to decreased body weight (Fig. 4C) and had abnormal lifespans (Fig. 4D). By the age of 8 months, the mortality rate in 492stop/Y mice reached 80%, while in the WT group, only one mouse died (from rectal prolapse). These developmental disabilities and significantly reduced life expectancy in 492stop/Y mice may be closely related to the chronic and imperceptible burden of spontaneous seizures. This is consistent with the shortened life expectancy reported in CDD patients, as most reported cases do not live beyond the median age range of 40–45 years [52].

### Altered Spine Density in the *Cdkl5* 492stop/Y Mouse Model

CDKL5 plays a crucial role in neuronal maturation, dendritic growth, synaptic activity, and dendritic spine morphogenesis, as demonstrated in numerous studies [11, 12, 47, 53–59]. However, findings regarding the impact of CDKL5 deficiency on pyramidal neuron morphology have been inconsistent [47, 53, 54, 60]. To address this, we analyzed dendritic spine morphology in adult 492stop/Y mice, focusing on pyramidal neurons in the CA1 region of the hippocampus, the somatosensory cortex, and Purkinje cells in the cerebellum.

**Fig. 5:**
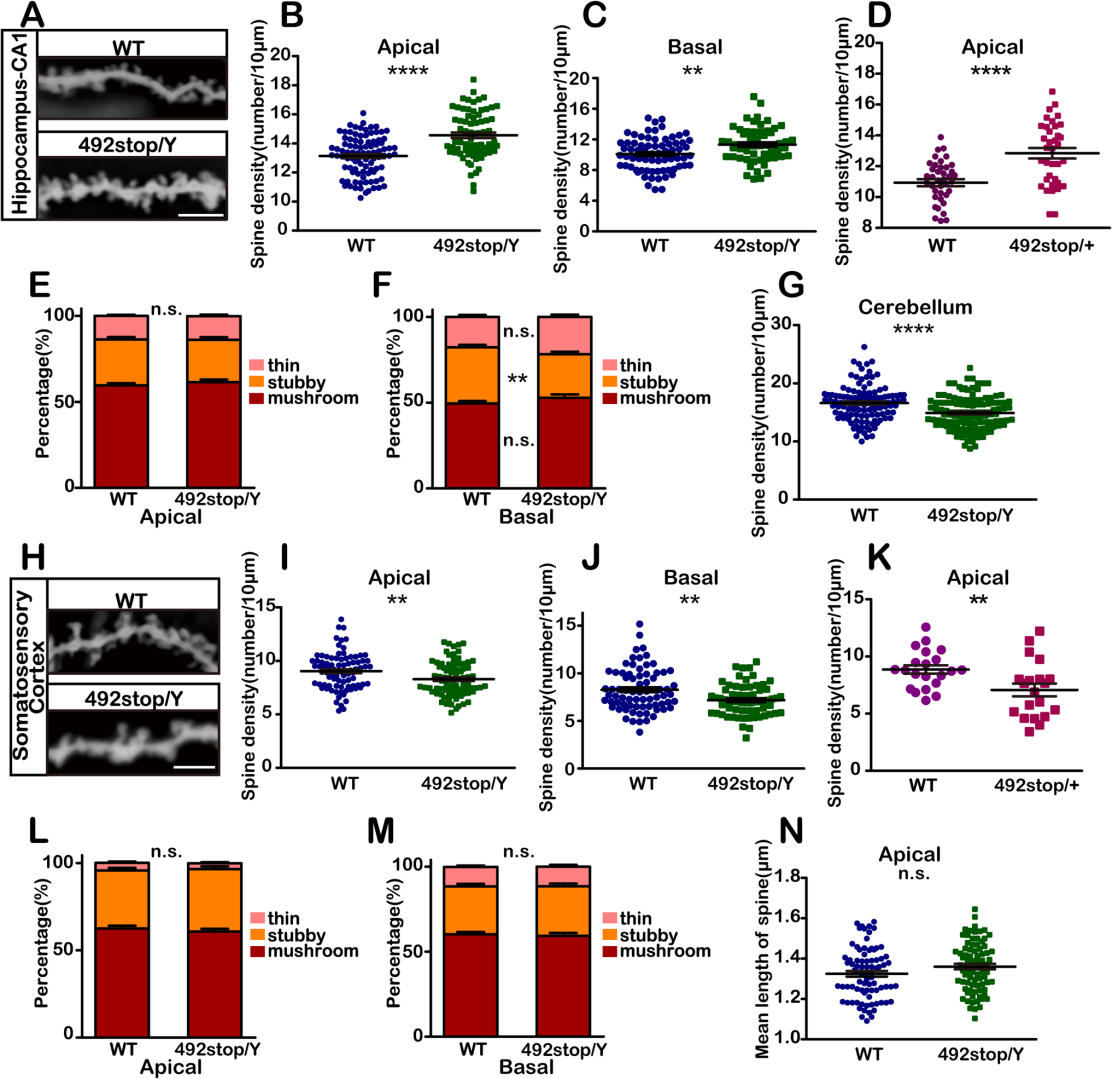
Altered spine density in 492stop/Y mice. **A**-**C**, **E**, **F** Spine density in pyramidal neurons in the hippocampal CA1 region in 4-month-old 492stop/Y mice and WT littermates. *n* >50 neurons per genotype, *n* ≥4 mice per genotype. **A** Representative images of Golgi-stained dendrites. Scale bar, 5 μm. **B**, **C** 492stop/Y mice have a significantly higher spine density than WT controls. **P <0.01, ****P <0.0001, unpaired two-tailed Student's *t-*test. **D** Spine density in female mice. Apical dendrites of pyramidal neurons in the hippocampal CA1 region in 4-month-old mice. *n* ≥35 neurons per genotype. ****P <0.0001, unpaired two-tailed Student's *t*-test. **E**, **F** Quantification of dendritic spine subtypes. The spine shape is divided into mushroom, stubby, and thin. Mushroom and stubby shapes are considered mature spines. n.s.: no significant difference, **P <0.01, two-way ANOVA, Sidak's multiple comparisons test. **G** Spine density in cerebellar Purkinje cells in 4-month-old 492stop/Y mice and WT littermates. *n* ≥100 neurons per genotype, *n* ≥4 mice per genotype. ****P <0.0001, unpaired two-tailed Student's *t*-test. **H**-**J**, **L**- **N** Pyramidal neurons in layers II/III of the somatosensory cortex in 4-month-old 492stop/Y mice and their WT littermates. *n* >50 neurons per genotype, *n* ≥4 mice per genotype. **H** Representative images of Golgi-stained dendrites. Scale bar, 5 μm. **I**, **J** 492stop/Y mice have significantly reduced spine density compared with WT controls. **P <0.01, unpaired two-tailed Student's *t*-test. **K** Spine density in female mice. Apical dendrites of layers II/III pyramidal neurons in 4-month-old mice. *n* ≥20 neurons per genotype. 492stop/+ mice have significantly lower spine density than WT controls. **P <0.01, unpaired two-tailed Student's *t*-test. **L**, **M** Quantification of dendritic spine subtypes. n.s.: no significant difference, two-way ANOVA, Sidak's multiple comparisons test. **N** Mean length of mushroom dendritic spines in 492stop/Y mice and their WT littermates. n.s.: no significant difference, unpaired two-tailed Student's *t-*test. All data are presented as the mean ± SEM.

In CA1 pyramidal neurons, both apical and basal spine densities were significantly increased by 12.4% and 10.9%, respectively, in 492stop/Y mice compared to WT controls (Fig. 5A-C). In heterozygous mice, random X-inactivation further exaggerated these changes, with a 17.5% increase (Fig. 5D). Interestingly, while the number of basal spines increased, there was a corresponding reduction in the proportion of mature spines, suggesting that CDKL5 is critically involved in spine maturation and impacts synaptic functional plasticity (Fig. 5E-F). This increase in spine density is consistent with findings from *Cdkl5*-KO and Nex-cre mice [47, 56, 57], indicating a possible link to heightened excitatory synaptic activity in CA1 pyramidal neurons [56].

Conversely, dendritic spine density in Purkinje cells decreased significantly by 10.3% in 492stop/Y mice compared to controls (Fig. 5G). This reduction aligns with previous reports of decreased inhibitory neurotransmitter release in CDKL5-deficient animals, leading to an imbalance in excitatory and inhibitory synaptic activity [55].

In layers II/III of the somatosensory cortex, apical and basal spine densities decreased by 8.2% and 13.2% in 492stop/Y mice, and by 20.2% in heterozygous 492stop/+ mice (Fig. 5H-K), accompanied by slight changes in spine maturity (Fig. 5L, M) and mushroom spine length (Fig. 5N). This reduction in spine density, found in both male hemizygotes and female heterozygotes, aligns with previous studies [58, 60, 61] and correlates with the behavioral deficits characteristic of intellectual disability in 492stop/Y mice [62].

As a neuronal activity-regulated molecule, CDKL5 influences synaptic plasticity by modulating the morphological changes of dendritic spines. It responds to signals from different brain regions to precisely control spine development and maturation through changes in protein interactions and substrate phosphorylation at synapses.

### Unusual Protein Degradation in C-Terminal Truncating Mutations Leads to CDKL5 Deficiency

Understanding the pathogenic mechanisms of C-terminal truncations in CDKL5 requires considering the reduction in CDKL5 protein levels. While previous studies have mainly focused on *Cdkl5* R59X mice, which mimic CDD patients with nonsense mutations in the N-terminal catalytic domain, the impact of C-terminal truncations remains less understood.

**Fig. 6:**
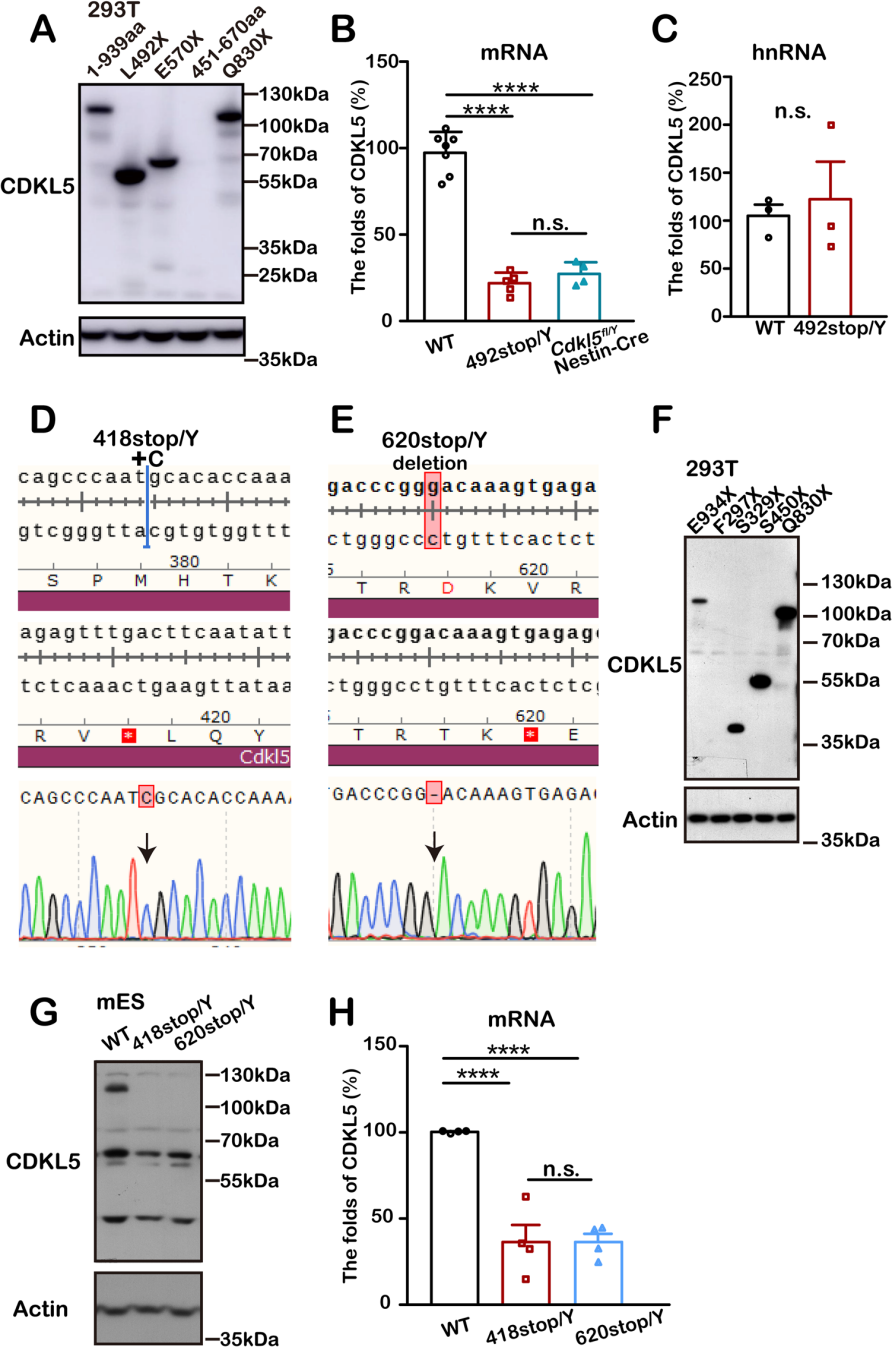
CDKL5 loss in the truncated mutants. **A** Western blots showing truncated CDKL5 protein from plasmid-based constructs *in vitro*, using an N-terminal CDKL5 antibody. **B**, **C** CDKL5 levels: mRNA (**B**) and hnRNA (**C**), normalized to GAPDH. **B** WT controls: *n =* 6, 492stop/Y: *n =* 5, Nestin-Cre: *n =* 4, ****P <0.0001, n.s. no significant difference; one-way ANOVA with Tukey's multiple comparisons. **C** WT controls: *n =* 3, 492stop/Y: *n =* 3, n.s. no significant difference; unpaired two-tailed Student's *t*-test. **D**, **E** Gene editing designs for generating 418stop/Y (**D**) and 620stop/Y (**E**) cell lines, confirmed by sequencing. **F** Western blots showing truncated CDKL5 from plasmid constructs *in vitro*, using an antibody targeting residues aa 300-600. **G** Western blots showing loss of full-length and truncated CDKL5 in 418stop/Y and 620stop/Y lines using the antibody from (**F)**. Truncated D418X and V620X are predicted to be 50 kDa and 74 kDa, respectively. **H** *Cdkl5* mRNA levels in 418stop/Y (*n =* 4) and 620stop/Y (*n =* 4) lines compared to untreated cells (*n =* 4), normalized to GAPDH. ****P <0.0001, n.s.: no significant difference; one-way ANOVA with Tukey’s multiple comparisons. All data are presented as the mean ± SEM.

A key question is whether C-terminal defects cause protein degradation. While endogenous CDKL5 protein loss was found in 492stop/Y mice (Fig. 2D), *in vitro* CDKL5 constructs of various lengths, including L492X, were normally expressed (Fig. 6A). As for mRNA levels, 492stop/Y mice showed a 78% *Cdkl5* reduction compared to WT controls (Fig. 6B), similar to the pattern seen in Nestin-Cre *Cdkl5*-KO mice. Interestingly, the level of hnRNA, representing the precursor form of mRNA, remained unchanged (Fig. 6C). These results suggest that protein loss in 492stop/Y mice is likely due to abnormal degradation rather than impaired *Cdkl5* mRNA synthesis, contributing to the CDKL5 deficiency and associated abnormal phenotypes found in 492stop mice.

### Decreased CDKL5 Expression in Cell Lines with C-Terminal Truncating Mutations

To avoid interference from specific mutations or background in the mouse model, we generated cell lines with various *Cdkl5* truncations. CRISPR/Cas9 editing in mouse embryonic cells induced mutations, resulting in CDKL5 mutants with different C-terminal truncation sites (Fig. 6D, E).

We first assessed CDKL5 expression in 418stop/Y and 620stop/Y cell lines (named after their premature stop sites). Consistent with 492stop/Y mice, truncated CDKL5 protein could not be detected at its expected sizes of 50 kDa and 74 kDa, after confirming the antibody's specificity (Fig. 6F, G). Furthermore, RT-PCR analysis showed a 64% reduction in *Cdkl5* mRNA levels in 418stop/Y and 620stop/Y cells compared to unmodified embryonic cells (Fig. 6H). These findings confirm that CDKL5 loss occurs not only in 492stop mice but also in these mutant cell lines. Thus, truncating mutations lead to complete CDKL5 deficits rather than partial protein defects.

### *Cdkl5* is Regulated by the Nonsense-Mediated RNA Decay (NMD) Pathway

The NMD pathway is a crucial mechanism for RNA quality control, rapidly degrading abnormal mRNAs with premature termination codons (PTCs) [63]. This degradation leads to gene inactivation and loss of function, providing a possible explanation for CDKL5 deficiency [64, 65].

To explore this, we first examined whether the *Cdkl5* truncating mutations conform to the 50-55 nucleotide rule [66, 67], which states that only PTCs located at least 50-55 nucleotides upstream of the final exon-exon junction can trigger NMD. All of the truncating mutations discussed above fell within this regulatory scope of NMD, satisfying the rule's requirements.

**Fig. 7:**
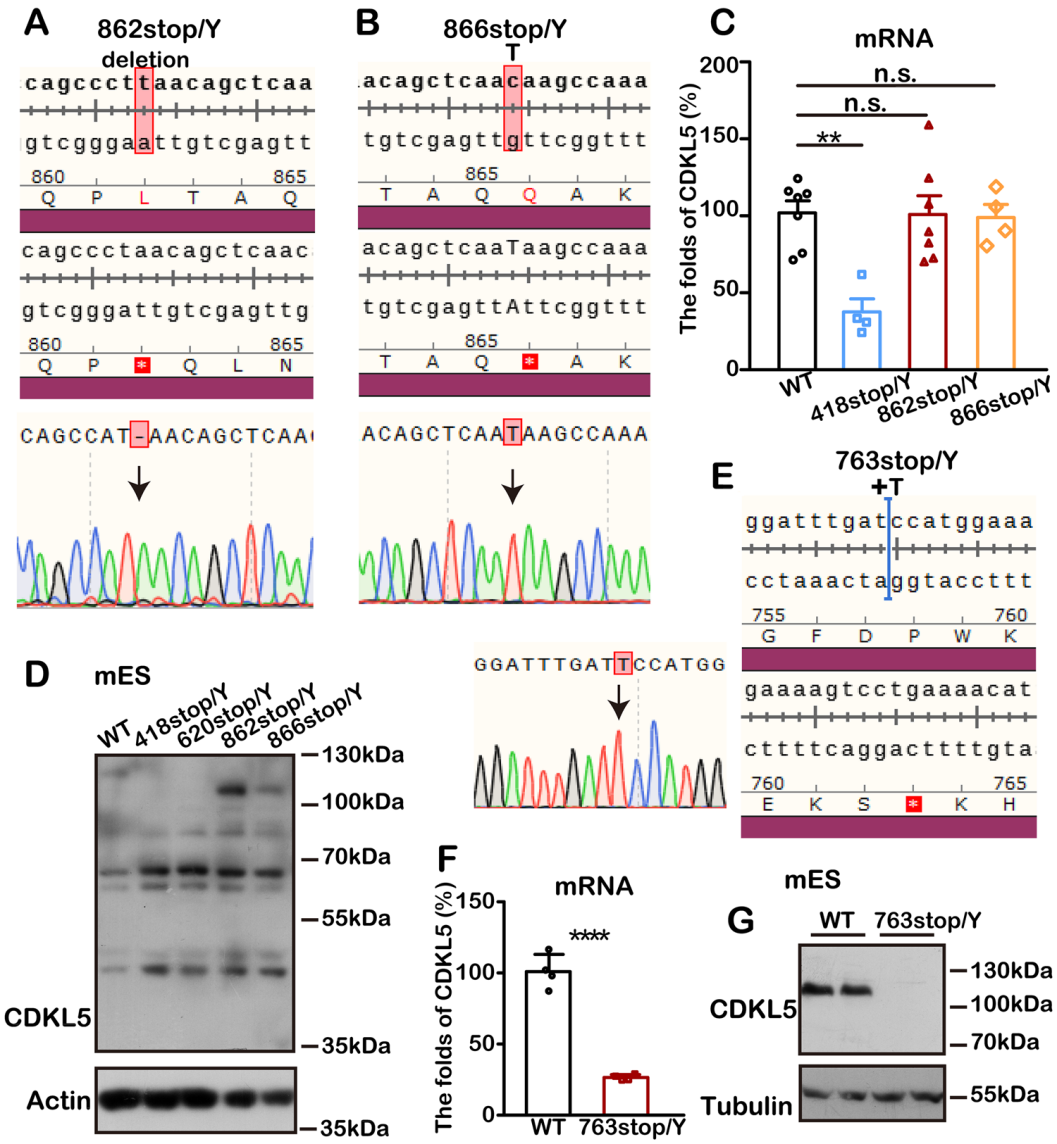
Loss of CDKL5 follows the regulatory rules of the NMD pathway. **A**, **B**, **E** Gene editing designs for constructing the 862stop/Y, 866stop/Y, and 763stop/Y cell lines. The constructs are verified by sequencing PCR products.** C**, **D** The expression of truncated CDKL5 is not reduced when the mutants are not predicted to be regulated by the NMD pathway. **C** Compared to the WT controls (*n =* 7), *Cdkl5* mRNA is not reduced in 862stop/Y (*n =* 7) and 866stop/Y (*n =* 4) cells, which differ from other mutations (418stop/Y: *n =* 4) under NMD supervision. All amplifications are normalized by GAPDH. **P <0.01, n.s.: no significant difference, one-way ANOVA with Tukey’s multiple comparisons test. **D** Western blots showing truncated CDKL5 proteins in 862stop/Y and 866stop/Y cells. Full-length CDKL5 is 113 kDa; 862stop/Y and 866stop/Y are 104 kDa. **F** The relative mRNA level is reduced in 763stop/Y (*n =* 4) cell lines. All amplifications are normalized by GAPDH. The WT group: *n =* 4, ****P <0.0001, unpaired two-tailed Student *t*-test. **G** Western blots show that truncated CDKL5 protein is absent from the 763stop/Y cell line. The predicted protein size is 91 kDa. All data are presented as the mean ± SEM.

Thus, we generated new cell lines, 862stop/Y and 866stop/Y (Fig. 7A, B), in which *Cdkl5* was predicted to escape NMD regulation. As expected, the levels of *Cdkl5* mRNA in the 862stop/Y and 866stop/Y cells remained comparable to WT controls (Fig. 7C), and truncated endogenous CDKL5 proteins were detected (Fig. 7D).

In contrast, the PTCs in 763stop/Y (Fig. 7E), located just 100 amino-acids (two exons) upstream of the 862stop, resulted in CDKL5 loss (Fig. 7F, G), providing direct evidence of NMD-mediated CDKL5 regulation. Notably, this cell line represents the termination site closest to the NMD regulatory limit in CDKL5, highlighting the pathway's sensitivity in regulating CDKL5 expression.

### CDKL5 Expression Changes upon Disruption of the NMD Pathway

**Fig. 8:**
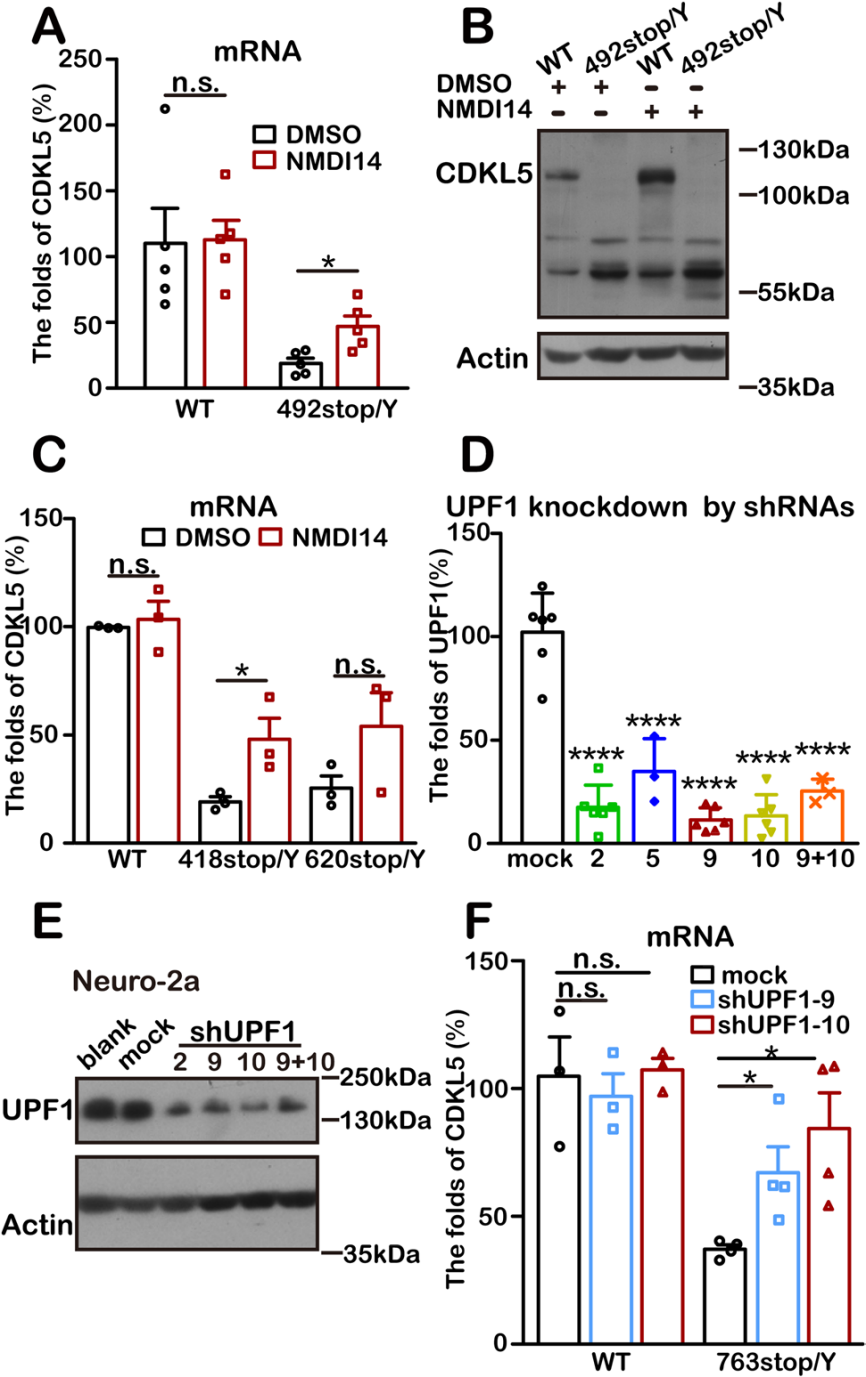
Truncated CDKL5 expression is increased by inhibition of the NMD pathway. **A**, **B** Cortical neurons cultured at P0 and NMDI14 treatment starts at DIV5. **A** The relative *Cdkl5* mRNA levels in 492stop/Y neurons (*n =* 5) increase after NMDI14 treatment compared to WT neurons (*n =* 5). All amplifications are normalized by RPL19. *P <0.05, n.s.: no significant difference, unpaired two-tailed Student *t*-test. **B** Western blots showing truncated CDKL5 protein (54 kDa) in 492stop/Y neurons after NMDI14 treatment. **C** The relative *Cdkl5* mRNA levels in 418stop/Y (*n =* 3) and 620stop/Y (*n =* 3) cells increase after NMDI14 treatment compared to WT controls (*n =* 3). All amplifications are normalized by RPL19. *P <0.05, n.s.: no significant difference, unpaired two-tailed Student *t*-test. **D** *Upf1* mRNA expression is repressed by RNAi. Each shUPF1 group differs from the mock (*n =* 6), with shUPF1-9 (*n =* 6) and shUPF1-10 (*n =* 6) showing the best efficiency in UPF1 knockdown. All amplifications are normalized by RPL19. shUPF1-2: *n =* 6, shUPF1-5: *n =* 3, shUPF1-9+10: *n =* 3; ****P <0.0001, one-way ANOVA with Tukey’s multiple comparisons test. **E** Western blots showing reduced UPF1 expression after RNAi. **F** The relative *Cdkl5* mRNA levels increase with UPF1 knockdown. All amplifications are normalized by RPL19. WT group: *n =* 3; 763stop/Y: *n =* 3; *P <0.05, n.s.: no significant difference, unpaired two-tailed Student *t*-test. All data are presented as the mean ± SEM.

To investigate the impact of disrupting the NMD pathway on CDKL5 expression, we treated 492stop/Y neurons with NMDI14, an NMD inhibitor that increases the expression of endogenous NMD targets [68]. NMDI14 treatment raised the *Cdkl5* mRNA levels from 19% to 47% compared to the DMSO-treated group, in contrast to the unchanged levels in the WT groups (Fig. 8A). Western blot analysis detected a half-sized CDKL5 protein following NMD pathway inhibition (Fig. 8B). Similarly, mutant cell lines also exhibited increased *Cdkl5* mRNA levels after NMDI14 treatment (Fig. 8C).

In addition to using the inhibitor, we disrupted the NMD pathway by targeting the main complex. We suppressed UPF1, a key NMD component [69, 70], with plasmid-based RNAi constructs. Among the tested shRNAs, shUPF1-9 and shUPF1-10 were the most effective in reducing UPF1 expression (Fig. 8D, E). As shown in Fig. 8F, transient transfection of shUPF1 in the 763stop/Y cell line significantly increased *Cdkl5* transcripts, similar to the effect of NMDI14 treatment.

These findings further confirm that CDKL5 is regulated by the NMD pathway. Truncating mutations with PTCs upstream of amino-acid 763, including at that site, cause CDKL5 protein degradation, leading to the deficiency in individuals with CDD.

## Discussion

In our study, we successfully generated a mouse model with a truncating mutation closely resembling premature termination codons found in CDD patients. Mutant transcript instability from mRNA surveillance and nonsense-mediated decay leads to CDKL5 protein loss, resulting in disease-related phenotypes in 492stop/Y mice. CDD is characterized by early-onset seizures, severe intellectual disability, and autism-like symptoms. Consistent with other knockout mice, 492stop/Y mice exhibit motor impairments, increased anxiety, reduced sociability, and deficits in learning and memory, along with altered neuronal morphology.

Importantly, our data provide compelling evidence that CDKL5 deficiency in 492stop/Y mice leads to spontaneous seizures, a feature rarely reported in male whole-body CDKL5-deficient mice. However, the limited detection of seizure-like activity underscores the challenges in identifying such events, likely due to discrepancies in genetic backgrounds and knockout strategies across laboratories. In CDD patients, early infantile epileptic encephalopathy is a core diagnostic criterion, typically manifesting within the first 3 months of life [6]. However, symptoms in 492stop/Y mice appear in adulthood or even in senescence. In addition to the undeniable differences between the patient and mouse model, this delayed onset may be due to a compensatory response to CDKL5 deficiency that begins during embryonic development and is passed down through generations. Furthermore, as infant mice cannot undergo EEG surgery, the identification and confirmation of weaker phenotypes and lower frequencies of early epilepsy are more challenging based solely on video observations.

To date, strategies to generate *Cdkl5*-KO mice have primarily focused on exon deletions or the R59X mutation, resulting in CDKL5 loss [7, 14, 57, 71]. Therefore, our 492stop mice, mimicking numerous cases with C-terminal truncating mutations, provide a valuable tool for studying neurological disorders and exploring therapeutic avenues for CDD.

Our study demonstrates that *Cdkl5* is regulated by the NMD pathway, with truncating mutations leading to protein loss when premature termination codons are upstream of amino-acid 763. Notably, several reported cases are consistent with our findings in mice. The mutant mRNA levels of human CDKL5 also exhibit reduced stability, indicating NMD regulation, such as the G632X and Q834X mutations found in female patients [4, 41].

**Fig. 9:**
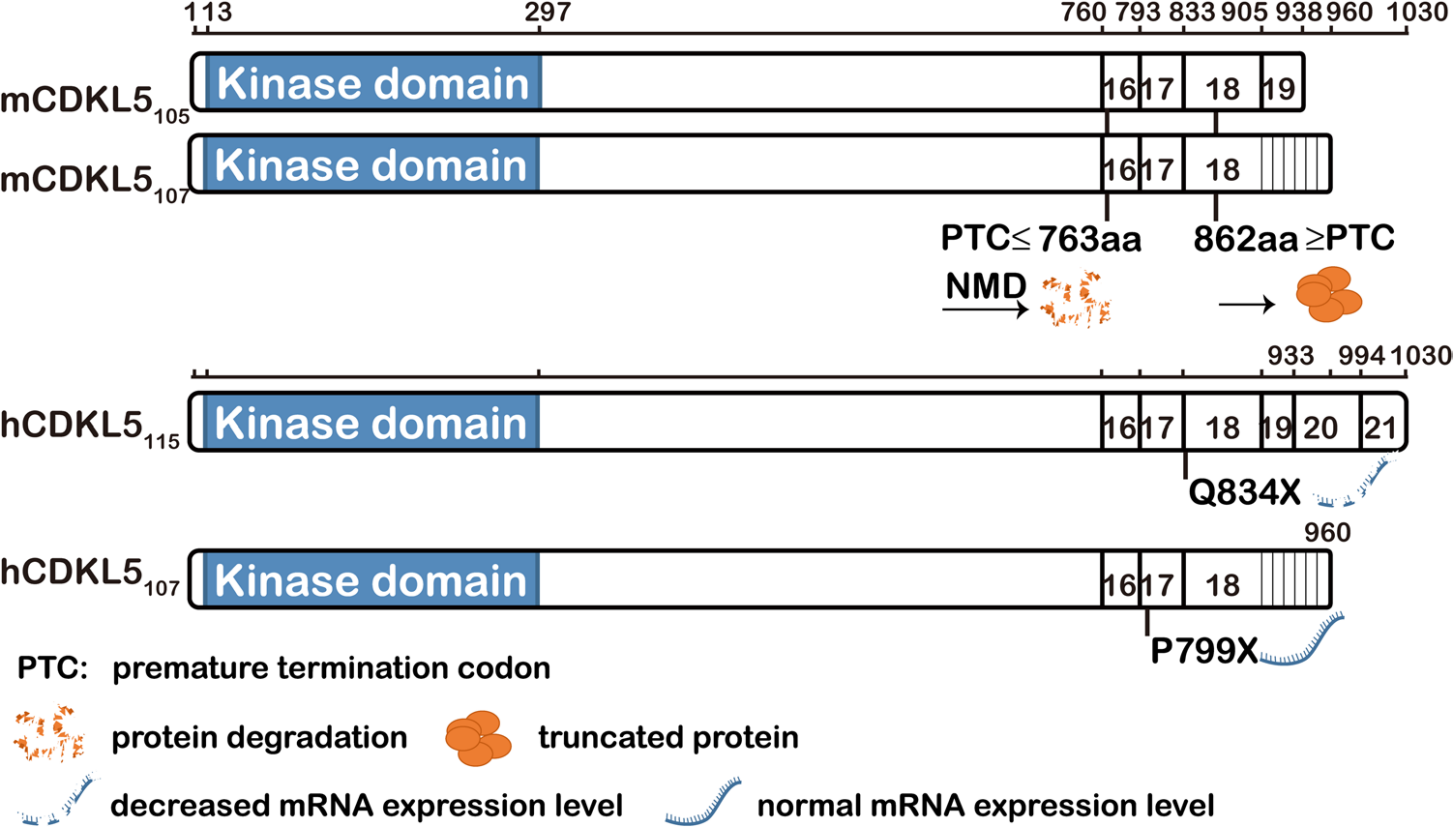
Schematics of truncated CDKL5 expression from different variants. In mice, truncating mutations lead to protein loss when PTCs are upstream of aa 763. In humans, differing escape regions result in undetectable Q834X from hCDKL5115 in lymphoblastoid cells [41], while P799X from hCDKL5107 escapes NMD and remains stable in fibroblasts [4].

In addition, the location of the PTC is crucial for NMD, with potential escape more likely at the last two exons [4, 41, 66, 72]. In mice, the common CDKL5 transcript variants (Fig. 9) share coding sequences except for the last exon [72]. Thus, truncated proteins likely arise when PTCs occur downstream of aa 793 (mCDKL5_107_) or aa 833 (mCDKL5_105_), consistent with sequencing results detecting both transcripts in 862stop/Y and normal cell lines (data not shown).

The NMD regulatory range differs in humans, with the original isoform hCDKL5_115_ producing a 1030 amino-acid protein and potential escape starting at exon 20 (aa 933). Therefore, truncated proteins that degrade *via* NMD in mice are also likely to degrade in humans. Nearly 85% of pathogenic mutations in hCDKL5_115_ have PTCs upstream of aa 763, correlating with over one-third of CDD patients. In addition, some reported pathogenic mutations are in isoform hCDKL5_107_ [72, 73], which has 18 exons and thus has distinct potential escape regions, possibly explaining the normal *Cdkl5* mRNA levels of the P799X mutation [4].

The existence and function of truncated CDKL5 proteins remain debated. Our study demonstrates that truncated CDKL5 mutants are subjected to the NMD pathway, with a small portion escaping it to express truncated CDKL5 [74]. Damage to the C-terminal tail disrupts CDKL5 function by altering its subcellular localization and interfering with protein-protein interactions [75, 76]. However, most truncated variants may not be present *in vivo*. Patients with truncating variants in a similar range exhibit varying severity [5, 16, 72, 77], probably due to mosaicism resulting in diverse proportions and distributions of defective cells [78, 79]. These findings underscore the importance of CDKL5 and indicate that even minor deletions in the C-terminal tail can lead to abnormal phenotypes in CDD patients. Overall, our results illuminate the pathological role of CDKL5 deficiency and provide insights into potential therapeutic interventions for CDD.

## Supplementary Information

Below is the link to the electronic supplementary material.

Supplementary file1 (PDF 112 kb)

Supplementary file2 (MP4 7123 kb)
