# Supplementary material for: A Novel Mouse Model Unveils Protein Deficiency in Truncated CDKL5 Mutations
Source: Neurosci Bull. 2025 Mar 5;41(5):805–20. doi: 10.1007/s12264-024-01346-4 (PMC12014890; doi:10.1007/s12264-024-01346-4)
Supplement: Supplementary file 1 — Supplementary file1 (PDF 112 kb) [file 12264_2024_1346_MOESM1_ESM.pdf]

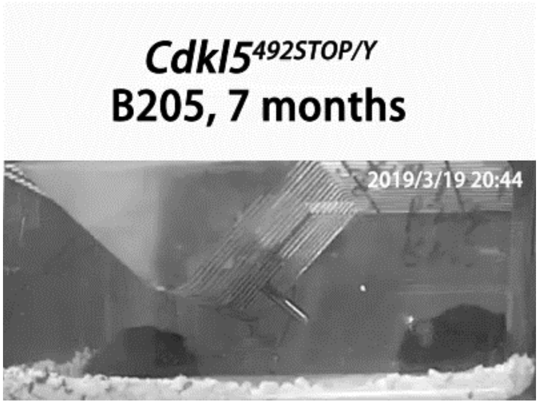

Video: The fatal spontaneous seizures in 492stop/Y mice. This 36-second video recorded the death images of a 7-month-age 492stop/Y mouse. Prior to the death, this mouse began some milder seizure-like activities such as Straub tail and head nodding, and rapidly progressed to complete a loss of postural control.
